# Incidence of persistent SARS-CoV-2 gut infection in patients with a history of COVID-19: Insights from endoscopic examination

**DOI:** 10.1055/a-2180-9872

**Published:** 2024-01-05

**Authors:** Mohamed Hany, Eman Sheta, Ahmed Talha, Medhat Anwar, Mohamed Selima, Muhammad Gaballah, Ahmed Zidan, Mohamed Ibrahim, Ann Samy Shafiq Agayby, Anwar Ashraf Abouelnasr, Mohamed Samir, Bart Torensma

**Affiliations:** 1 54562 Department of Surgery, Medical Research Institute, Alexandria University, Hadara, Alexandria 21561, Egypt; 2 54562 Department of Pathology, Alexandria University, Alexandria, Egypt; 3 4501 Epidemiology, Leiden University Medical Center, Leiden, Netherlands

**Keywords:** Endoscopy Upper GI Tract, Epidemiology

## Abstract

**Background and study aims** Gut infection is common during acute COVID-19, and persistent SARS-CoV-2 gut infection has been reported months after the initial infection, potentially linked to long-COVID syndrome. This study tested the incidence of persistent gut infection in patients with a history of COVID-19 undergoing endoscopic examination.

**Patients and methods** Endoscopic biopsies were prospectively collected from patients with previous COVID-19 infection undergoing upper or lower gastrointestinal endoscopy (UGE or LGE). Immunohistochemistry was used to detect the presence of persistent SARS-CoV-2 nucleocapsid proteins.

**Results** A total of 166 UGEs and 83 LGE were analyzed. No significant differences were observed between patients with positive and negative immunostaining regarding the number of previous COVID-19 infections, time since the last infection, symptoms, or vaccination status. The incidence of positive immunostaining was significantly higher in UGE biopsies than in LGE biopsies (37.34% vs. 16.87%, *P* =0.002). Smokers showed a significantly higher incidence of positive immunostaining in the overall cohort and UGE and LGE subgroups ( *P* <0.001). Diabetic patients exhibited a significantly higher incidence in the overall cohort ( *P* =0.002) and UGE subgroup ( *P* =0.022), with a similar trend observed in the LGE subgroup ( *P* =0.055).

**Conclusions** Gut mucosal tissues can act as a long-term reservoir for SARS-CoV-2, retaining viral particles for months following the primary COVID-19 infection. Smokers and individuals with diabetes may be at an increased risk of persistent viral gut infection. These findings provide insights into the dynamics of SARS-CoV-2 infection in the gut and have implications for further research.

## Introduction

The COVID-19 pandemic, caused by the novel coronavirus "severe acute respiratory syndrome coronavirus 2" (SARS-CoV-2), began as an outbreak of pneumonia in December 2019. By March 2020, the World Health Organization had declared it a global pandemic 1 . The implications have been profound, resulting in substantial life loss and significant economic crises worldwide. New cases and deaths related to COVID-19 continue to be reported 2 . COVID-19 extends beyond the respiratory system, with documented effects on other systems, notably the gastrointestinal tract. More than 10% of patients have reported symptoms such as nausea, vomiting, and diarrhea during acute illness, occasionally preceding the onset of respiratory symptoms 3 4 5 6 7 8 9 . In addition, viral RNA has been detected in the feces of approximately 50% of COVID-19 patients, regardless of gastrointestinal symptoms. In vitro models have shown that human-entered cells can host productive SARS-CoV-2 infections 10 11 12 .

Evidence shows that viral particles can persist in different human tissues, including neural, cardiac, adipose, and gut tissues, for an extended period after primary COVID-19. This persistence has been associated with the phenomenon known as "long COVID-19" 5 9 13 14 15 16 17 18 .

Immunohistochemistry has been effectively utilized to detect SARS-CoV-2-infected cells by identifying viral proteins within the cytoplasm of these cells 5 9 13 16 17 19 . Despite this, there are limited data regarding persistent SARS-CoV-2 gut infection and the patient characteristics associated with this persistence or the development of long COVID-19 syndrome. Consequently, this study aimed to assess the incidence of persistent SARS-CoV-2 infection in the gut cells of patients with the history of COVID-19. These assessments were performed using immunohistochemistry during endoscopic examinations at a high-volume endoscopic unit, and the results were used to compare the characteristics of patients with positive and negative immunostaining.

## Patients and methods

This was a random patient selection in a cross-sectional study design conducted between October 2022 and February 2023. Patients undergoing upper gastrointestinal endoscopy (UGE) or colonoscopy (lower gastrointestinal endoscopy [LGE]) during the study period were asked about their history of COVID-19. Those with polymerase chain reaction (PCR)-confirmed COVID-19 were invited to participate in the study. Participants provided informed consent for the use of their data in future research. The study was approved by our institutional Ethics Committee and adhered to the 1964 Declaration of Helsinki.

### Endpoints

The primary endpoint was detection of Persistent SARS-CoV-2 gastrointestinal infection gut infection using immunohistochemistry in endoscopic biopsies from patients with a history of COVID-19 9 20 . The secondary endpoints were comparison of immunostaining results between UGE and colonoscopy; assessment of the association between positive immunostaining and clinical factors, including number of previous COVID-19, time since last infection, symptoms of initial COVID-19, and vaccination status; and analysis of risk factors associated with positive immunostaining.

### Inclusion criteria

Patients were eligible if they had a history of PCR-confirmed COVID-19, with or without gastrointestinal symptoms, at least 1 month before endoscopy. They also had to exhibitno active illness indicative of COVID-19, including respiratory and gastrointestinal symptoms. All patients exhibiting only clinical symptoms or suspicions with mild or high fever were subjected to PCR testing and were advised to return home to await the results, ensuring the absence of COVID-19 during endoscopic biopsy.

### Data collection

Data included demographic information, associated medical problems, original pathology in the endoscopic biopsies, COVID-19 serology tests at the time of endoscopy, vaccination information (type, number of doses, side effects), time elapsed since last vaccine dose, breakthrough infection and details about previous COVID-19 (symptoms, number of episodes, and the duration between the last illness and the time of endoscopy).

### Tissue collection and examination

Endoscopic punch biopsies for viral particle detection were randomly taken from healthy-appearing mucosa in the stomach, colon, and terminal ileum using cold biopsy forceps. Biopsies were fixed in 10% buffered formalin for 24 hours, embedded in paraffin, and sectioned into 5-µm slices with a Leica RM2235 rotary microtome. The slices were mounted on positively charged slides and underwent 3,3'-diaminobenzidine (DAB) immunohistochemistry using an automated Dako autostainer (LINK 48). After antigen retrieval using EDTA solution for 15 minutes at pH 9, the slides were incubated with an anti-SARS-CoV-2 nucleocapsid monoclonal mouse immunoglobulin-G (IgG) antibody (Bio-techne, USA, #MAB10474–100) at a 1:1000 concentration for 30 minutes. After adding freshly prepared chromogen (DAB) for 2 minutes and counterstaining with hematoxylin, the stained slides were assessed for positivity 13 .

### Quantification of SARS-CoV-2 antibody response

The serological response to SARS-CoV-2 was measured using an enzyme-linked immunosorbent assay (ELISA_-based SARS-CoV-2 IgG assay (AnshLabs, Webster, Texas, United States) that quantifies antibodies to spike and nucleocapsid proteins. The Dynex automated analyzer was used to calculate the antibody concentration in arbitrary concentration units (AU/mL). A concentration of >12 AU/mL was considered positive, <10 AU/mL negative, and 10 to 12 AU/mL was considered an indeterminate 21 .

### Statistical analysis

For the analyses, we used descriptive and inferential statistics. All data were first tested for normality using the Kolmogorov-Smirnov, Q-Q plot, and Levene’s tests. Categorical variables were expressed as n (%). Continuous normally distributed variables were described with their means and standard deviations, while non-normally distributed variables were expressed with their medians and interquartile ranges. When appropriate, categorical variables were tested using Pearson’s Chi-squared or Fisher’s exact test. Continuous normally distributed data were tested with the student’s *t* -test for independent samples. For non-normally distributed data, the Mann-Whitney U-test was used for independent samples.

All independent variables counting more than 10 events and showing *P* <0.1 were eligible for multivariable analysis, achieved through backward selection. The estimated effects from the model were presented as adjusted odds ratios (AORs) and 95% confidence intervals (95% CI) to represent the likelihood of positivity associated with each variable. An AOR greater than 1 indicates a higher risk of positivity, whereas an AOR less than 1 suggests a lower risk. Statistical significance was set at *P* ≤0.05. Data were analyzed using R software version 4.2.2 (The R Project for Statistical Computing, Vienna, Austria).

## Results

This cross-sectional study included 249 patients with a median age of 53 (range 14 to 88 years). Males represented 52% of the cohort. Hypertension and diabetes mellitus (DM) were the most commonly observed associated medical conditions at 14.5% and 12.4%, respectively. Of the biopsies undertaken, 67% were from the upper gastrointestinal tract and 33% were from the lower gastrointestinal tract. The lower gastrointestinal tract samples comprised both colon and ileum biopsies per patient, accounting for 95%.

Regarding SARS-CoV-2 findings, nucleocapsid proteins of the virus were identified in 76 biopsies, accounting for 31%. The stomach was the predominant site of these findings, with 82% of the identified proteins. Of the biopsies, 89% showed no general pathological abnormalities, but gastritis was evident in 12 patients, which is 4.8%. Antibodies specific to SARS-CoV-2 (IgG) were detected in 32.5% of the patients, translating to 81 individuals. The median antibody titer among these was 36.8 AU/mL. SARS-CoV-2 nucleocapsid tissue staining was discernible in gastric biopsies from 37.34% of patients for cytoplasmic staining.

**Fig. 1:**
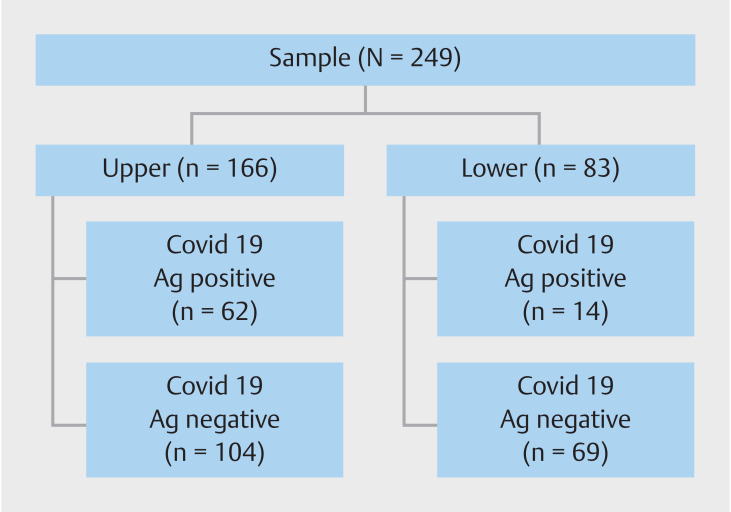
Distribution of positive and negative immunostaining for cytoplasmic SARS-CoV-2 nucleocapsid proteins in endoscopic biopsies.

**Fig. 2:**
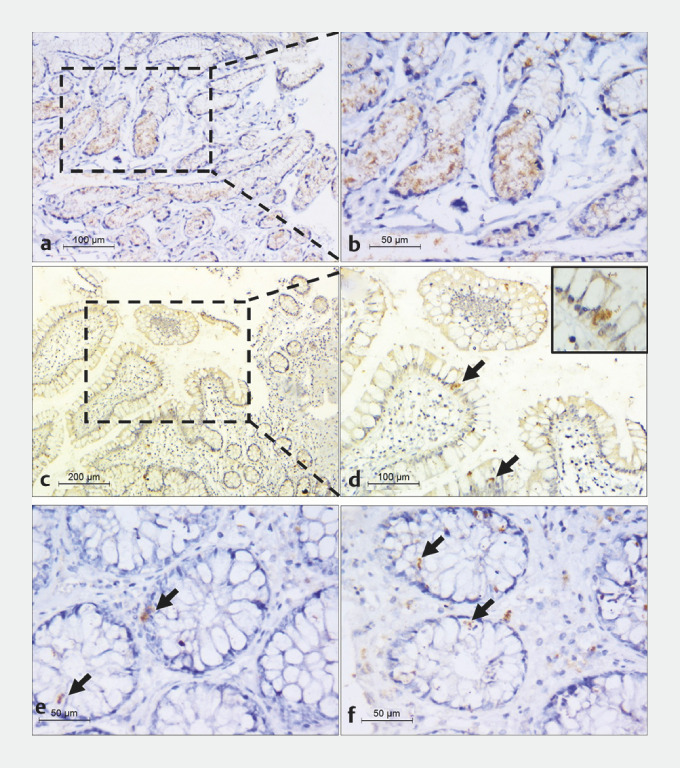
Sections from endoscopic biopsies stained by anti-SARS-Cov2 nucleocapsid antibodies. **a** Gastric biopsy showing positive staining in gastric glands (x200). **b** A higher-power view of the gastric biopsy shows fine granular staining in the cytoplasm (x400). **c** An ileal biopsy showing focal positive staining (x100). **d** Higher magnification showing positive staining of epithelial cells (arrows) (x200), inset highlights cytoplasmic positivity (x400). **e,f** Focal positive staining in two colonic biopsies (arrows) (x400).

In addition, colonic and ileal biopsies from 16.87% of patients displayed this staining. In gastric biopsies, the staining at the crypt base manifested as fine brownish cytoplasmic granules. In contrast, the colonic and ileal biopsies presented focal staining within the epithelial cells. Despite the positive staining, the histology was predominantly normal, though there was a mild increase in the lymphoplasmacytic infiltrate ( Table 1 , Fig. 1 , Fig. 2 ).

**Table:** **Table 1** Patient characteristics.

Variable	N	%
Male sex	130	52.2
Age (years), median, min-max	53	14–88
Anthropometrics		
Height, median, min-max	1.66	1.48–1.85
Weight, median, min-max	75	40–122
BMI, median, min-max	27.2	15.6–48.9
Smoking status	41	16.5
Comorbidities		
HTN	36	14.5
DM	31	12.4
Asthma	14	5.6
cardiac	4	1.6
Dyslipidemia	4	1.6
Biopsy		
Type of endoscopy		
Upper	166	66.7
Lower	83	33.3
Site of biopsy		
Stomach	166	66.7
Colon and ileum	79	31.7
Colon	4	1.6
Positive stain	76	30.5
Site of positive stain		
Stomach	62	81.6
Colon & ileum	12	15.8
Ileum	2	2.6
Pathology if present		
None	222	89.2
Gastritis	12	4.8
Adenoma	6	2.4
Colitis	3	1.2
Adenocarcinoma	2	0.8
Adenoma with dysplasia	1	0.4
Hyperplastic polyp	1	0.4
Reactive gastropathy	1	0.4
Ulcerative colitis	1	0.4
COVID-19 IgG, median, min - max	36.8	0.1–216.3
BMI, body mass index; HTN, hypertension; DM, diabetes mellitus; IgG, immunoglobulin G		

### History of previous COVID-19

The median duration since the last infection was reported as 17 months, with a range spanning 7 to 30 months. Of the patients, 79% had experienced a single COVID-19 episode. One hundred eight (43%) reported gastrointestinal symptoms related to their infection. The primary symptoms were loss of smell/taste (41%), followed by nausea (26%) and diarrhea (18%). Regarding vaccination, 172 patients (69%) had received an anti-COVID-19 vaccine. Sinovac was the most common vaccine administered, accounting for 51%, with Pfizer being the second most common at 32%. Among those vaccinated, 72% had completed their vaccination with at least two doses. Notably, 127 subjects (91.5%) reported no side effects post-vaccination ( Table 2 ).

**Table:** **Table 2** History of COVID-19 infection and vaccination.

Variable	N	%
History of COVID-19 infection		
Time elapsed since the last infection	17	7–30
Number of past infections		
One	197	79.1
Two	37	14.9
Three	15	6.0
COVID-19 symptoms		
Fever	98	39.4
Malaise	125	50.2
Cough	40	16.1
Sore throat	69	27.7
Dyspnea	21	8.4
ICU	1	0.4
Any GI symptoms	108	43.4
Loss of smell/taste	103	41.4
Nausea	65	26.1
Diarrhea	44	17.7
Abdominal pain	14	5.6
Vomiting	19	7.6
Vaccinated cases	172	69.1
Vaccine type		
AstraZeneca	26	15.1
Pfizer	55	32.0
Sinopharm	2	1.2
Sinovac	89	51.7
Number of doses		
One	11	6.4
Two	123	71.5
Three	38	22.1
Time elapsed since last vaccine dose, Median, min - max	12	(9, 21)
Breakthrough infection	44	26.7
Vaccine side effects		
No reported side effects	157	91.3
Fever	6	3.5
Fever and malaise	3	1.8
Malaise	2	1.2
Abdominal pain and cough	1	0.6
Fever and arrhythmia	1	0.6
Fever and cough	1	0.6
Fever injection site hotness	1	0.6
ICU, intensive care unit; GI, gastrointestinal		

### Characteristics of patients with positive and negative SARS-CoV-2

In the entire cohort, biopsies that tested positive were significantly more frequent among UGE patients (37.3%) compared to LGE patients (16.8%; *P* =0.002). The distribution of negative SARS-CoV-2 results was 62.6% in UGE and 83.1% in LGE, respectively. Among patients with positive staining, smoking (36.8% vs. 7.5%) and DM (22.3% vs. 8.1%) were significantly associated when compared to those with negative staining ( *P* <0.001 and *P* =0.002, respectively) ( Table 3 ).

**Table:** **Table 3** COVID-19 Ag-positive and -negative cases in the whole cohort.

**Variable**	**Positive** (N= 76)	**Negative** (N= 173)	***P* value **
Male	41 (53.9)	89 (51.4)	.821
Age, median (min–max)	54 (15–80)	52 (14–88)	.739
Anthropometrics			
Height, median (min-max)	1.68 (1.5–1.85)	1.66 (1.48–1.82)	.836
Weight, median (min-max)	79 (45–120)	75 (40–122)	.209
BMI, Median (min-max)	27.7 (17.2–40.0)	26.3 (15.6-–48.9)	.292
Smoking status	28 (36.8)	13 (7.5)	<0.001*
Comorbidities			
HTN	14 (18.4)	22 (12.7)	0.326
DM	17 (22.4)	14 (8.1)	0.002*
Asthma	3 (3.9)	11 (6.4)	0.561
Cardiac	0 (0.0)	4 (2.3)	0.317
Dyslipidemia	1 (1.3)	3 (1.7)	1.000
Biopsy type			0.002*
Lower	14 (18.4)	69 (39.9)	
Upper	62 (81.6)	104 (60.1)	
Site of biopsy			
Stomach	62 (81.6)	104 (60.1)	0.003*
Colon/ileum	14 (18.4)	65 (37.6)	
Colon	0 (0.0)	4 (2.3)	
History of COVID-19 infection			
Number of infections			0.866
One	61 (80.3)	136 (78.6)	
Two	10 (13.2)	27 (15.6)	
Three	5 (6.6)	10 (5.8)	
Time elapsed since the last infection	17 (7–29)	17 (7–30)	0.927
COVID-19 symptoms			
Fever	31 (40.8)	67 (38.7)	0.868
Malaise	33 (43.4)	92 (53.2)	0.200
Sore throat	8 (10.5)	32 (18.5)	0.165
Cough	26 (34.2)	43 (24.9)	0.172
Dyspnea	4 (5.3)	17 (9.8)	0.323
ICU	1 (1.3)	0 (0.0)	0.305
GIT symptoms	33 (43.4)	75 (43.4)	1.000
Abd pain	32 (42.1)	71 (41.0)	0.986
Nausea	11 (14.5)	33 (19.1)	0.486
Vomiting	4 (5.3)	10 (5.8)	1.000
Diarrhea	21 (27.6)	44 (25.4)	0.836
Loss smell taste	7 (9.2)	12 (6.9)	0.716
Vaccinated cases	54 (71.1)	118 (68.2)	0.765
vaccine type			0.639
AstraZeneca	6 (11.1)	20 (16.9)	
Pfizer	17 (31.5)	38 (32.2)	
Sinopharm	1 (1.9)	1 (0.8)	
Sinovac	30 (55.6)	59 (50.0)	
number of doses			0.650
One	2 (3.7)	9 (7.6)	
Two	39 (72.2)	84 (71.2)	
Three	13 (24.1)	25 (21.2)	
Time elapsed since last vaccine dose, Median (Min, Max)	12 (9, 21)	12 (9, 20)	0.728
Breakthrough infection	12 (22.6)	32 (28.6)	0.538
COVID-19 IgG, Median (min–max)	40.2 (0.1–143.2)	35.1 (0.2–216.3)	0.357
* Statistically significant results ≤0.05. HTN, hypertension; DM, diabetes mellitus; ICU, intensive care unit; IgG, immunoglobulin G.			

Stratification on UGE and LGE showed that the incidence of smoking was observed in patients with positive staining (33.8% vs. 10.5%) ( *P* <0.001); DM was significantly more present in UGE (22.5% vs. 8.7%) ( Table 4 , Table 5 ).

**Table:** **Table 4** COVID-19 Ag-positive and -negative upper GIT biopsies.

Variable n=(%)	Positive (N=62)	Negative (N=104)	*P* value
Male sex	34 (54.8)	54 (51.9)	0.839
Age	53 (15–80)	50 (14–88)	0.480
Anthropometrics			
Height	1.66 (1.5–1.85)	1.65 (1.5–1.82)	0.736
Weight	79 (45–120)	70 (40–105)	0.023
BMI	27.8 (17.2–40)	25.8 (15.6–41.0)	0.046
Smoking status	21 (33.9)	11 (10.6)	<0.001*
Comorbidities			
HTN	11 (17.7)	15 (14.4)	0.728
DM	14 (22.6)	10 (8.7)	0.022*
Asthma	3 (4.8)	4 (3.8)	1.000
Cardiac	0 (0.0)	1 (1.0)	1.000
Dyslipidemia	1 (1.6)	2 (1.9)	1.000
History of COVID-19 infection			
Number of infections			0.517
One	48 (77.4)	85 (81.7)	
Two	9 (14.5)	15 (14.4)	
Three	5 (8.1)	4 (3.8)	
Time elapsed since the last infection	17 (7–27)	18 (7–30)	0.280
COVID-19 Symptoms			
Fever	24 (38.7)	45 (43.3)	0.679
Malaise	26 (41.9)	56 (53.8)	0.185
Sore throat	8 (12.9)	18 (17.3)	0.593
Cough	21 (33.9)	22 (21.2)	0.104
Dyspnea	4 (6.5)	8 (7.7)	1.000
ICU	1 (1.6)	0 (0.0)	0.373
GIT symptoms	27 (43.5)	42 (40.4)	0.812
Abd pain	30 (48.4)	36 (34.6)	0.112
Nausea	7 (11.3)	19 (18.3)	0.329
Vomiting	4 (6.5)	5 (4.8)	0.729
Diarrhea	19 (30.6)	28 (26.9)	0.736
Loss smell taste	6 (9.7)	6 (5.8)	0.367
Vaccinated cases	42 (67.7)	68 (65.4)	0.888
vaccine type			0.925
AstraZeneca	4 (9.5)	9 (13.2)	
Pfizer	13 (31.0)	23 (33.8)	
Sinopharm	1 (2.4)	1 (1.5)	
Sinovac	24 (57.1)	35 (51.5)	
number of doses			0.524
One	1 (2.4)	5 (7.4)	
Two	31 (73.8)	50 (73.5)	
Three	10 (23.8)	13 (19.1)	
Time elapsed since last vaccine dose, Median (Min, Max)	12 (9, 21)	12 (9, 20)	0.977
Breakthrough infection	10 (24.4)	16 (24.6)	1.000
COVID-19 IgG	42.9 (0.1–143.2)	42.5 (0.2–151.6)	0.766
*Statistically significant results ≤0.05. HTN, hypertension; DM, diabetes mellitus; ICU, intensive care unit; IgG, immunoglobulin G.			

**Table:** **Table 5** COVID-19 Ag-positive and -negative lower GIT biopsies.

Variable n=(%)	Positive (N=14)	Negative (N= 69)	*P* value
Male sex	7 (50.0)	35 (50.7)	1.000
Age	56 (17–73)	55 (17–81)	0.784
Anthropometrics			
Height	1.70 (1.50–1.80)	1.66 (1.48–1.8)	0.431
Weight	75.5 (50–95)	75 (45–122)	0.435
BMI	26.1 (19.0–33.7)	27.5 (17.6–48.9)	0.293
Smoking status	7 (50)	2 (2.9%)	<0.001*
Comorbidities			
HTN	3 (21.4)	7 (10.1)	0.361
DM	3 (21.4)	4 (5.8)	0.055
Asthma	0 (0.0)	7 (10.1)	0.596
Cardiac	0 (0.0)	3 (4.3)	1.000
Dyslipidemia	0 (0.0)	1 (1.4)	1.000
History of COVID-19 infection			
Number of previous COVID-19 infections			0.443
One	13 (92.9)	51 (73.9)	
Two	1 (7.1)	12 (17.4)	
Three	0 (0.0)	6 (8.7)	
Time elapsed since the last infection	19 (8–29)	16 (7–30)	0.065
COVID-19 Symptoms			
Fever	7 (50.0)	22 (31.9)	0.323
Malaise	7 (50.0)	36 (52.2)	1.000
Sore throat	0 (0.0)	14 (20.3)	0.112
Cough	5 (35.7)	21 (30.4)	0.756
Dyspnea	0 (0.0)	9 (13.0)	0.345
ICU	6 (42.9)	33 (47.8)	0.777
GI symptoms	2 (14.3)	35 (50.7)	0.017
Abd pain	4 (28.6)	14 (20.3)	0.491
Nausea	0 (0.0)	5 (7.2)	0.583
Vomiting	2 (14.3)	16 (23.2)	0.724
Diarrhea	1 (7.1)	6 (8.7)	1.000
Loss smell taste	12 (85.7)	50 (72.5)	0.482
Vaccinated cases	7 (50.0)	22 (31.9)	0.323
vaccine type			1.000
AstraZeneca	2 (16.7)	11 (22.0)	
Pfizer	4 (33.3)	15 (30.0)	
Sinopharm	0 (0.0)	0 (0.0)	
Sinovac	6 (50.0)	24 (48.0)	
number of doses			1.000
One	1 (8.3)	4 (8.0)	
Two	8 (66.7)	34 (68.0)	
Three	3 (25.0)	12 (24.0)	
Time elapsed since last vaccine dose, median (min, max)	14 (9, 20)	12 (9, 19)	0.357
Breakthrough infection	2 (16.6)	16 (34.0)	0.311
COVID-19 IgG	25.5 (7.3–121.5)	23.3 (0.3–216.3)	0.189
*Statistically significant results ≤0.05. BMI, body mass index; HTN, hypertension; DM, diabetes mellitus; ICU, intensive care unit; GI, gastrointestinal; IgG, immunoglobulin G.			

### Multiple logistic regression analysis

In the logistic regression analysis assessing factors influencing the likelihood of a positive stain test result, several predictors were examined: age, sex, DM, smoking status, type of endoscopy, number of previous COVID-19 infections, time since the last infection, and vaccination status. Smoking status showed a pronounced effect, with an odds ratio (OR) of 7.68 (95% CI: 3.56, 17.46, *P* <0.001), signifying an increased likelihood of a positive test among smokers. The type of endoscopy was another significant predictor; patients undergoing upper endoscopy were more likely to test positive than those with lower endoscopy, with an OR of 2.74 (95% CI: 1.38, 5.72, *P* =0.005). DM was significantly associated with a positive test result, demonstrated by an OR of 2.84 (95% CI: 1.17, 6.99, *P* =0.021). In the segmented analysis focusing on patients with prior vaccination, we examined additional factors: the type of vaccine, number of doses received, time since the last vaccine dose, and history of breakthrough COVID-19 post-vaccination. None of these factors significantly influenced the likelihood of a positive stain test result ( Table 6 ).

**Table:** **Table 6** Logistic regression analysis for predicting the probability of positive stain results.

Term	OR	SE	95% CI	*P* value
(Intercept)	0.07	0.84	(0.01, 0.33)	0.001*
Age	1.01	0.01	(0.99, 1.03)	0.321
Sex (male vs female)	0.95	0.32	(0.51, 1.80)	0.886
DM	2.84	0.45	(1.17, 6.99)	0.021*
Smoking	7.68	0.40	(3.56, 17.46)	<0.001*
Type of endoscopy (Upper vs lower)	2.74	0.36	(1.38, 5.72)	0.005*
No of infections	1.37	0.27	(0.79, 2.32)	0.242
time since infection	0.99	0.03	(0.93, 1.04)	0.605
Vaccinated vs non-vaccinated	1.03	0.36	(0.51, 2.10)	0.934
Segmented analysis on vaccinated individuals				
Vaccine type				
AstraZeneca vs inactivated	0.37	0.64	(0.10, 1.23)	0.124
Pfizer vs inactivated	0.71	0.43	(0.30, 1.63)	0.420
Number of vaccine doses	1.39	0.39	(0.64, 3.03)	0.408
Time elapsed since vaccination	1.10	0.07	(0.96, 1.25)	0.161
Breakthrough infection	0.38	0.62	(0.11, 1.28)	0.125
OR, odds ratio; SE, standard error; CI, confidence interval; DM, diabetes mellitus. *Significant (P <.05).				

## Discussion

This study had 249 cross-sectional patients with UGE or colonoscopy (LGE), whereby samples for SARS-CoV-2 infection were tested.

Evidence indicates active SARS-CoV-2 replication in enterocytes, leading to gut infection in acute COVID-19 cases 3 4 5 6 7 8 9 10 11 12 . Consequently, stringent protective measures are recommended during endoscopic procedures, especially for patients with COVID-19, to prevent viral transmission to healthcare providers 22 . The susceptibility of gastrointestinal cells to SARS-CoV-2, due to ACE2 receptor expression, results in severe gut involvement during acute COVID-19, with gastrointestinal bleeding rates of 2% to 13% in hospitalized patients 3 5 6 7 8 .

### Gastrointestinal manifestations during acute COVID-19

A multicenter study by Vanella et al. elucidated endoscopic findings in patients with COVID-19, with 44.4% experiencing gastrointestinal symptoms. Predominantly, ischemic-like colitis was observed in colonoscopies, whereas gastro-duodenal ulcers and erosions were frequent in UGE 3 . Similarly, Mauro et al. identified ulcers as the most common finding in UGE for upper gastrointestinal bleeding in patients with acute COVID-19 23 . Moreover, Massironi et al. reported bulbar ulcers, gastric erosions, esophagitis, and ischemic colitis as principal findings in endoscopic examinations of acute COVID-19 patients 24 . Neuberger et al. reported severe erosive duodenitis in 8% of critically ill, intensive care unit-admitted patients with COVID-19 who had pneumonia and were experiencing severe gastrointestinal symptoms, such as bleeding or feeding intolerance. These symptoms were directly attributed to enterocyte infection by SARS-CoV-2, as confirmed by positive immunostaining for the virus's spike protein in endoscopic biopsies 5 . The virus's direct invasion of the endothelial epithelium, which expresses ACE-2 receptors, could elucidate the ischemic gastrointestinal lesions and general thrombotic events observed in acute COVID-19 3 .

### Potential pathophysiological pathways

SARS-CoV-2, responsible for COVID-19, uses the angiotensin-converting enzyme 2 (ACE2) receptor to enter cells. These ACE2 receptors are abundant not just in the respiratory tract, but also in the small intestine's enterocytes, making the gastrointestinal system a target for infection 25 26 . The virus can directly damage the gastrointestinal epithelium, manifesting in symptoms like diarrhea. Notably, viral particles have been detected in feces even when negative respiratory tests indicate potential prolonged gastrointestinal infection or viral shedding 6 27 . The immune response to the virus can also cause local inflammation in the gut, increasing its barrier permeability and potentially leading to or worsening diarrhea 28 . In addition, there is a notable connection between the gut and lungs, known as the "gut-lung axis." Any disruption in the gut can influence respiratory health and vice versa 29 . Systemically, the virus's inflammatory response, especially releasing cytokines like interleukin-6, can affect gastrointestinal physiology 30 .

Lastly, there are reports from some "long-haulers" who experience extended gastrointestinal symptoms after the primary infection phase has passed, indicating potential lasting effects on the gastrointestinal system. However, the exact causes are still under study 31 .

### Persistent gut infection by SARS-CoV-2

Persistent gut infection by SARS-CoV-2 has been reported, with viral RNA and nucleocapsid proteins detected in surgical samples from the stomach, colon, intestine, gallbladder, bile, and peritoneal fluid months post-infection 13 14 15 . These viral components have also been identified in endoscopic biopsies from the small bowel and colon long after COVID-19 resolution 5 9 16 17 .

This study detected SARS-CoV-2 nucleocapsid proteins in 30.5% of patients, primarily in the UGE subgroup (37.34%). Only 43.4% of these patients had prior gut involvement in their COVID-19. Immunohistochemistry confirmed persistent infection of the gut. Despite biopsies being from seemingly healthy mucosa, an increased lymphoplasmacytic infiltrate, indicative of a potent immune response, was seen in samples with positive immunostaining.

This study underscores the relatively common occurrence of persistent SARS-CoV-2 in the gut post-COVID-19, even without acute gastrointestinal symptoms, aligning with prior research identifying the gut as a long-term SARS-CoV-2 reservoir 9 . Arostegui et al. identified a case of enduring SARS-CoV-2 in the colon 3 months post-infection, linked with chronic abdominal pain and elevated fecal calprotectin. This persistent viral colonization, evidenced by positive immunohistochemical staining for SARS-CoV-2 nucleocapsid proteins and dense lymphocytic infiltrations in colonic biopsies, may suggest a sustained immune response and potential etiology for gastrointestinal-dominant long COVID-19 17 .

Zollner et al. detected SARS-CoV-2 nucleocapsid proteins and RNA in endoscopic biopsies from patients with inflammatory bowel disease several months post-acute COVID-19 resolution 16 . Similarly, Cherne et al. studied SARS-CoV-2 persistence in endoscopic gastrointestinal biopsies and fluid samples, employing PCR, immunohistochemical staining, and virus isolation assays. The study included 100 patients with unknown prior COVID-19 infection and 12 with confirmed infection. Results showed a low incidence of persistent SARS-CoV-2 gut infection—only one biopsy from the unknown infection status group and three from the confirmed group tested positive. Moreover, no viable SARS-CoV-2 virions were isolated from the samples, and the virus and its RNA were found to be inactivated entirely within 24 hours of exposure to colonic fluids 9 .

The inability to isolate infectious virions from gut tissues and fluids and their rapid inactivation suggest these persistent particles pose a minimal infection risk to healthcare professionals performing endoscopic procedures. However, persistent viral particles have been associated with "long COVID-19" syndrome 15 16 18 , characterized by lingering symptoms post-primary COVID-19 32 . Affecting 30% to 87% of COVID-19 patients 17 , long COVID-19 can manifest various gastrointestinal symptoms, including anorexia, dysphagia, bowel motility changes, abdominal pain, and weight loss 18 .

### Long COVID-19 syndrome

The gastrointestinal variant of long COVID-19 syndrome, characterized by persistent viral colonization leading to ongoing inflammation and cellular abnormalities, has been extensively documented 15 16 17 18 33 34 35 36 . Our study identified a higher incidence of persistent SARS-CoV-2 infection, as indicated by positive immunostaining, among smokers and individuals with DM. This difference was significant in the overall cohort and the subgroup undergoing UGE, suggesting smoking and DM could be a potential risk factor for developing long COVID-19 syndrome.

Although research on long COVID-19 risk factors is still limited, studies by Bai et al. 36 . Barthélémy et al. 37 , and Su et al. 38 have identified associations between long COVID-19 and factors such as smoking, older age, female gender, type 2 DM, SARS-CoV-2 RNAemia, Epstein-Barr virus viremia, and auto-antibodies during the initial COVID-19. Patients with these risk factors, especially those with DM, should receive close monitoring due to their increased susceptibility to long-term COVID-19 and potential exacerbation of acute illness.

Our study employed antibodies targeting nucleocapsid proteins to detect persistent SARS-CoV-2 virions using a well-established approach reported in previous research 5 9 13 16 17 19 . Immunohistochemistry staining for nucleocapsid and spike proteins exhibited higher sensitivity compared to RNA detection via reverse transcription polymerase chain reaction (RT-PCR), likely due to the increased stability of proteins. However, it should be noted that immunostaining was positive in only 75% of RT-PCR-positive gut mucosa samples in another study 16 .

In our cohort, 32.5% of patients displayed SARS-CoV-2 anti-spike IgG antibodies, with a median titer of 36.8 AU/mL. This antibody response could have stemmed from either prior infections or vaccinations. The median duration between the most recent infection and endoscopy was 7 months (range 3 to 30 months). In addition, the vaccination rate stood at 69%. There were no notable disparities in antibody prevalence, past infections, or vaccination status when comparing patients with positive and negative tissue staining results.

Our study employed an enzyme-linked immunosorbent assay (ELISA), which typically yields higher seroprevalence estimates than random access immunoassay (RAIA). Existing literature corroborates the persistence of a robust anti-spike IgG response for over a year post-infection 32 39 .

Furthermore, it is important to emphasize that our study did not utilize validated diagnostic criteria to test for long COVID-19 syndrome. There need to be more universally accepted and validated testing methods for long COVID-19. As such, our study did not specifically assess the identification and characterization of long COVID-19 in our cohort. Future research using validated diagnostic criteria for long COVID-19 syndrome will offer a more thorough understanding of its prevalence and related risk factors.

### Limitations

The absence of PCR testing in individuals for whom there was no suspicion of COVID-19 or who were symptom-free could have indicated missed infections during endoscopy; therefore, we cannot rule out the possibility of active subclinical COVID-19 in these cases. Another limitation is the need for follow-up data to monitor changes in persistent gut infection or disease progression over time in a patient. This study, being a cross-sectional design, has inherent limitations.

Follow-up has dual implications in this context. First, there is the duration over which we monitored for the development of persistent SARS-CoV-2 gastrointestinal infections. These results indicated that this duration was sufficient. Nevertheless, cross-sectional studies provide a snapshot of data at a specific time, restricting our ability to establish causality or capture changes over time. Consequently, the findings should be interpreted within the context of this study design.

Despite these limitations, the relatively large cohort from a single endoscopic center offers valuable insights. However, future multicenter studies with larger cohorts are warranted to validate the results further and minimize potential biases and confounding factors. Randomly selecting patients in this cohort reduces selection bias but limits the available variables, making complex modeling challenging. In addition, including longitudinal follow-up would provide a better understanding of the dynamics of persistent gut infection and its implications.

## Conclusions

This study underscores the relatively prevalent persistence of SARS-CoV-2 infection in gut cells after an initial COVID-19 episode, even in the absence of gastrointestinal symptoms. Smokers and patients with diabetes seem to be at an elevated risk of continuous viral gut infection and the subsequent onset of long COVID-19 syndrome. These observations emphasize the need for more in-depth research to understand better the mechanisms and clinical consequences of enduring gut infection and its correlation with protracted COVID-19 symptoms.

## References

[1] World Health Organization. WHO Director-General’s opening remarks at the media briefing on COVID-19 https://www.who.int/director-general/speeches/detail/who-director-general-s-opening-remarks-at-the-media-briefing-on-covid-19

[2] World Health Organization. Weekly epidemiological update on COVID-19 https://www.who.int/publications/m/item/weekly-epidemiological-update-on-covid-19

[3] Vanella G Capurso G Burti C Gastrointestinal mucosal damage in patients with COVID-19 undergoing endoscopy: an international multicentre study BMJ Open Gastroenterol 2021 8 e000578 10.1136/bmjgast-2020-000578 PMC7907837 33627313

[4] Brooks EF Bhatt AS The gut microbiome: a missing link in understanding the gastrointestinal manifestations of COVID-19? Cold Spring Harb Mol Case Stud 2021 7 a006031 10.1101/mcs.a006031 33593727 PMC8040733

[5] Neuberger M Jungbluth A Irlbeck M Duodenal tropism of SARS-CoV-2 and clinical findings in critically ill COVID-19 patients Infection 2022 50 1111 1120 10.1007/s15010-022-01769-z 35182354 PMC8857399

[6] Xiao F Tang M Zheng X Evidence for gastrointestinal infection of SARS-CoV-2 Gastroenterology 2020 158 1831 1.833E6 32142773 10.1053/j.gastro.2020.02.055 PMC7130181

[7] Lin L Jiang X Zhang Z Gastrointestinal symptoms of 95 cases with SARS-CoV-2 infection Gut 2020 69 997 1001 32241899 10.1136/gutjnl-2020-321013

[8] Martin TA Wan DW Hajifathalian K Gastrointestinal bleeding in patients with coronavirus disease 2019: a matched case-control study Am J Gastroenterol 2020 115 1609 1616 32796176 10.14309/ajg.0000000000000805 PMC7446989

[9] Cherne MD Gentry AB Nemudraia A Severe acute respiratory syndrome coronavirus 2 is detected in the gastrointestinal tract of asymptomatic endoscopy patients but is unlikely to pose a significant risk to healthcare personnel Gastro Hep Advances 2022 1 844 852 10.1016/j.gastha.2022.06.002 35765598 PMC9225937

[10] Wu Y Guo C Tang L Prolonged presence of SARS-CoV-2 viral RNA in faecal samples Lancet Gastroenterol Hepatol 2020 5 434 435 10.1016/S2468-1253(20)30083-2 32199469 PMC7158584

[11] Britton GJ Chen-Liaw A Cossarini F Limited intestinal inflammation despite diarrhea, fecal viral RNA and SARS-CoV-2-specific IgA in patients with acute COVID-19 Sci Rep 2021 11 13308 34172783 10.1038/s41598-021-92740-9 PMC8233421

[12] Zang R Castro MFG McCune BT TMPRSS2 and TMPRSS4 promote SARS-CoV-2 infection of human small intestinal enterocytes Sci Immunol 2020 5 eabc3582 32404436 10.1126/sciimmunol.abc3582 PMC7285829

[13] Hany M Zidan A Gaballa M Lingering SARS-CoV-2 in gastric and gallbladder tissues of patients with previous COVID-19 infection undergoing bariatric surgery Obes Surg 2023 33 139 148 36316598 10.1007/s11695-022-06338-9 PMC9628579

[14] Cheruiyot I Sehmi P Ngure B Laparoscopic surgery during the COVID-19 pandemic: detection of SARS-COV-2 in abdominal tissues, fluids, and surgical smoke Langenbecks Arch Surg 2021 406 1007 1014 33675407 10.1007/s00423-021-02142-8 PMC7936592

[15] Meringer H Mehandru S Gastrointestinal post-acute COVID-19 syndrome Nat Rev Gastroenterol Hepatol 2022 19 345 346 10.1038/s41575-022-00611-z 35383321 PMC8981882

[16] Zollner A Koch R Jukic A Postacute COVID-19 is characterized by gut viral antigen persistence in inflammatory bowel diseases Gastroenterology 2022 163 495 5.06E10 35508284 10.1053/j.gastro.2022.04.037 PMC9057012

[17] Arostegui D Castro K Schwarz S Persistent SARS-CoV-2 nucleocapsid protein presence in the intestinal epithelium of a pediatric patient 3 months after acute infection JPGN Reports 2022 3 e152 10.1097/PG9.0000000000000152 37168753 PMC10158423

[18] Mehandru S Merad M Pathological sequelae of long-haul COVID Nat Immunol 2022 23 194 202 10.1038/s41590-021-01104-y 35105985 PMC9127978

[19] Cheung CCL Goh D Lim X Residual SARS-CoV-2 viral antigens detected in GI and hepatic tissues from five recovered patients with COVID-19 Gut 2022 71 226 229 34083386 10.1136/gutjnl-2021-324280

[20] Sandal S Tanas Ö Karabulut Ş A histopathological view at the long-term effects of covid-19 on the gastrointestinal system in children: a single center experience Turk J Pediatr 2023 65 416 10.24953/turkjped.2022.1039 37395961

[21] Nguyen NN Mutnal MB Gomez RR Correlation of ELISA method with three other automated serological tests for the detection of anti-SARS-CoV-2 antibodies PLoS One 2020 15 e0240076 33022019 10.1371/journal.pone.0240076 PMC7537879

[22] Gralnek IM Hassan C Beilenhoff U ESGE and ESGENA Position Statement on gastrointestinal endoscopy and the COVID-19 pandemic Endoscopy 2020 52 483 490 10.1055/a-1155-6229 32303090 PMC7295280

[23] Mauro A De Grazia F Lenti MV Upper gastrointestinal bleeding in COVID-19 inpatients: Incidence and management in a multicenter experience from Northern Italy Clin Res Hepatol Gastroenterol 2021 45 101521 32888875 10.1016/j.clinre.2020.07.025 PMC7427596

[24] Massironi S Viganò C Dioscoridi L Endoscopic findings in patients infected with 2019 novel coronavirus in Lombardy, Italy Clin Gastroenterol Hepatol 2020 18 2375 2377 32480008 10.1016/j.cgh.2020.05.045 PMC7260560

[25] Zhou P Yang X-L Wang X-G A pneumonia outbreak associated with a new coronavirus of probable bat origin Nature 2020 579 270 273 32015507 10.1038/s41586-020-2012-7 PMC7095418

[26] Hoffmann M Kleine-Weber H Schroeder S SARS-CoV-2 cell entry depends on ace2 and tmprss2 and is blocked by a clinically proven protease inhibitor Cell 2020 181 271 2.8E10 32142651 10.1016/j.cell.2020.02.052 PMC7102627

[27] Lamers MM Beumer J Van Der Vaart J SARS-CoV-2 productively infects human gut enterocytes Science 2020 369 50 54 10.1126/science.abc1669 32358202 PMC7199907

[28] Zuo T Zhang F Lui GCY Alterations in gut microbiota of patients with COVID-19 during time of hospitalization Gastroenterology 2020 159 944 9.55E10 32442562 10.1053/j.gastro.2020.05.048 PMC7237927

[29] Budden KF Gellatly SL Wood DLA Emerging pathogenic links between microbiota and the gut-lung axis Nat Rev Microbiol 2017 15 55 63 27694885 10.1038/nrmicro.2016.142

[30] Mehta P McAuley DF Brown M COVID-19: consider cytokine storm syndromes and immunosuppression Lancet 2020 395 1033 1034 10.1016/S0140-6736(20)30628-0 32192578 PMC7270045

[31] Carfì A Bernabei R Landi F Persistent symptoms in patients after acute COVID-19 JAMA 2020 324 603 605 10.1001/jama.2020.12603 32644129 PMC7349096

[32] Raveendran AV Misra A Post COVID-19 syndrome (“Long COVID”) and diabetes: challenges in diagnosis and management: diabetes & metabolic syndrome Clin Res Rev 2021 15 102235 10.1016/j.dsx.2021.102235 PMC8317446 34384972

[33] Fändriks L The angiotensin II type 2 receptor and the gastrointestinal tract J Renin Angiotensin Aldosterone Syst 2010 11 43 48 10.1177/1470320309347788 19861352

[34] Lee S Yoon GY Myoung J Robust and persistent SARS-CoV-2 infection in the human intestinal brush border expressing cells Emerg Microbe Infect 2020 9 2169 2179 10.1080/22221751.2020.1827985 PMC7580600 32969768

[35] Jacobs JJL Persistent SARS-2 infections contribute to long COVID-19 Medical Hypotheses 2021 149 110538 10.1016/j.mehy.2021.110538 33621843 PMC7884250

[36] Bai F Tomasoni D Falcinella C Female gender is associated with long COVID syndrome: a prospective cohort study Clin Microbiol Infect 2022 28 6.11E11 6.11E18 34763058 10.1016/j.cmi.2021.11.002 PMC8575536

[37] Barthélémy H Mougenot E Duracinsky M Smoking increases the risk of post-acute COVID-19syndrome: Results from a French community-based survey Tob Induc Dis 2022 20 1 10 10.18332/tid/150295 PMC9204712 35799625

[38] Su Y Yuan D Chen DG Multiple early factors anticipate post-acute COVID-19 sequelae Cell 2022 185 881 8.95E22 35216672 10.1016/j.cell.2022.01.014 PMC8786632

[39] Masiá M Fernández-González M Telenti G Durable antibody response one year after hospitalization for COVID-19: A longitudinal cohort study J Autoimmunity 2021 123 102703 10.1016/j.jaut.2021.102703 34303083 PMC8289631

